# Efficacy of electroacupuncture in patients with failed back surgery syndrome: study protocol for a randomized controlled trial

**DOI:** 10.1186/s13063-021-05652-4

**Published:** 2021-10-14

**Authors:** Xiaoping Sheng, Hongyu Yue, Qi Zhang, Deta Chen, Weidong Qiu, Jun Tang, Tianyou Fan, Jingliang Gu, Bingchen Jiang, Minlei Qiu, Lin Chen

**Affiliations:** 1 Shanghai Municipal Hospital of Traditional Chinese Medicine, Shanghai University of Traditional Chinese Medicine, 274 Middle Zhijiang Road, Shanghai, 200071 China; 2 Shanghai Xuhui District Xietu Community Health Service Center, Shanghai, 200032 China

**Keywords:** Failed back surgery syndrome, Electroacupuncture, Manual acupuncture, Sham acupuncture, Clinical trial

## Abstract

**Background:**

Persistent pain following back surgery called failed back surgery syndrome remains a major treatment challenge. The aim of this study is to evaluate the efficacy and safety of electroacupuncture on relieving back pain in FBSS patients.

**Methods/design:**

This is a randomized, single-blind, single-site, placebo-controlled trial. A total of 144 eligible FBSS patients will be randomly assigned to the electroacupuncture, manual acupuncture, or sham acupuncture group in a 1:1:1 ratio. Each group will receive 2 treatment sessions per week for 12 weeks. The primary outcome will be low back pain intensity based on the 11-point numerical rating scale (NRS). The secondary outcomes include Oswestry Disability Index (ODI) questionnaire, Beck Depression Inventory-II (BDI-II), Pittsburgh Sleep Quality Index (PSQI), and analgesic consumption. All clinical outcomes will be collected at baseline, during the treatment phase (at 8 and 12 weeks), and at the 16-, 24- and 36-week follow-ups. All data will be analyzed based on the intention-to-treat principle and adverse events will be assessed during the trial.

**Discussion:**

This pilot randomized controlled trial will evaluate the efficacy of electroacupuncture for treating failed back surgery syndrome. The outcomes will determine whether electroacupuncture is efficacious in relieving low back pain as well as improving the quality of life in failed back surgery syndrome patients.

**Trial registration:**

Chinese Clinical Trial Registry ChiCTR2000040144. Registered on 22 November 2020

**Supplementary Information:**

The online version contains supplementary material available at 10.1186/s13063-021-05652-4.

## Background

“Failed back surgery syndrome” (FBSS) is characterized by persistent or recurrent back and/or radiating leg pain, even after anatomically successful spinal surgery [1]. Despite the advances made in lumber surgical technologies, the incidence of FBSS is still reported in the range of 10 to 40% [2]. Chronic pain following FBSS can always interfere with physical, emotional, and social components of life, resulting in a considerable financial burden on the society [3].

Due to the complex etiologies and mixed neuropathic and nociceptive pain components, few management guidelines existed for FBSS [4–6]. In general, FBSS without structural deficit was usually managed with conservative treatments, including oral medication, nerve blocks, pulsed radiofrequency, and physiotherapy [7, 8]. FBSS cases with poor response to conservative treatments are often treated with spinal cord stimulation (SCS) [9]. However, SCS requires implantation of a permanent pulse generator and the implant materials are expensive, which is not suitable for all medical conditions and patients. Therefore, finding the optimal management of FBSS as a therapy that was effective, less complicated, and not invasive in nature has been always desired.

Acupuncture is a therapeutic intervention originated in China, which can treat diseases by the insertion of thin metallic needles through the skin at specific sites [10]. In recent years, acupuncture has been used as an integrative therapy for pain since well-tolerated with little risk of adverse effects [11]. Numerous systematic reviews have investigated that acupuncture has a therapeutic effect on chronic low back pain [12, 13]. And the mechanisms of acupuncture analgesic effect were reported to involve endogenous pain control systems, cerebral plasticity, and nonspecific effects [14].

Electroacupuncture (EA) is the application of electrical stimulation to acupuncture needles. Several studies demonstrated that EA exerted a quicker and stronger analgesic effect than manual acupuncture [15, 16]. Furthermore, evidence-based results also showed that EA was better than high-frequency transcutaneous electrical nerve stimulation (TENS) or sham acupuncture in the treatment of neuropathic pain [17]. However, the efficacy of EA or acupuncture for FBSS has not been reported specifically. Therefore, we designed this rigorous high-quality randomized controlled trial to provide valid evidence for the efficacy of EA in treating FBSS, compared with manual acupuncture and sham acupuncture treatment.

## Methods

### Study design and setting

**Fig. 1:**
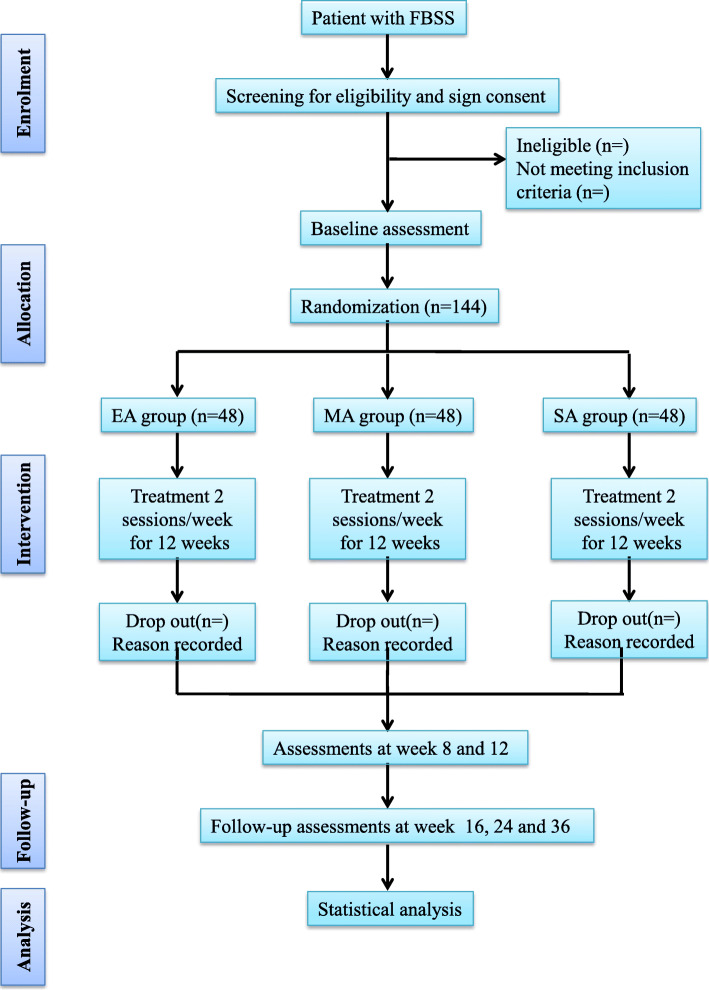
Flow chart of study procedures

This is a patient- and assessor-blind randomized, placebo-controlled clinical trial that will be conducted at Shanghai Municipal Hospital of Traditional Chinese Medicine. Eligible FBSS participants will be randomly assigned (1:1:1) to receive 24 sessions of electroacupuncture (EA), manual acupuncture (MA), or sham acupuncture (SA) over a 12-week treatment period and a 6-month follow-up period. The study will follow the recommendations of the Consolidated Standards of Reporting Trials (CONSORT) [18] and the protocol was reported in accordance with the Standard Protocol Items (SPIRIT) (Additional file 1). A flow chart of the study process is detailed in Fig. 1.

### Recruitment and ethics

The recruitment will be conducted at Shanghai Municipal Hospital of Traditional Chinese Medicine. Patients will be recruited throughout the patient clinic and hospital-based WeChat advertising. This study has been approved by the ethics committee of Shanghai Municipal Hospital of Traditional Chinese Medicine (No. 2020-SHL-KY-40), registered at the Chinese Clinical Trial Registry (ChiCTR2000040144), and will be carried out in accordance with the principles of the Declaration of Helsinki. All participants will be prioritized and asked to sign the informed consent before enrollment and have been given adequate time to consider what the trial involves. The study schedule of enrolment, intervention, and assessments is shown in Table 1.

**Table 1:**
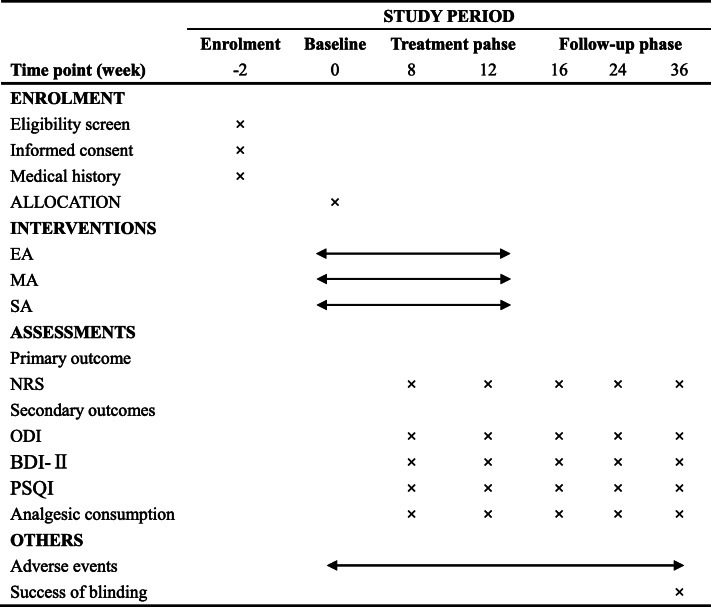
Schedule of enrollment, intervention, and assessments

### Inclusion criteria

Individuals will be recruited for the study if they satisfy the following criteria:

1. Aged 18–80 years

2. Primary diagnosis of FBSS [19] (in this study, FBSS is defined as low back and leg pain persist or recur for at least 6 months following at least one decompression and/or fusion procedure) and not requiring further surgery

3. Average low back pain score ≥6 on a 0–10 numerical rating scale (NRS)

4. On a stable dose of pain medications for at least 2 weeks prior to screening and willing to decrease the pain medication dose during the study

5. No evidence of failure to improve substantially with conservative management including but not limited to physical therapy, acupuncture, exercises, drug therapy, and bedrest

6. Willing to prohibit any treatment that may affect trial results other than acupuncture treatment

7. Competent to understand the study protocol and volunteer to write informed consent to participate

### Exclusion criteria

Individuals will be excluded if they have any of the following criteria:

1. Specific serious diseases that would be possible causes of spinal pain (e.g., malignant tumors, vertebral fractures, spinal infection, inflammatory spondylitis, cauda equina syndrome)

2. Consistent severe pain (10 out of 10 NRS) without fluctuation

3. Unsuitable for acupuncture or at risk of acupuncture-associated safety complications (e.g., hemorrhagic disease, patients under anticoagulant treatment, severe diabetes with risk of infection, serious cardiovascular or renal disease, skin lesions at the acupuncture site)

4. Medical conditions that would potentially influence the interpretation of treatment effect or results (e.g., diabetic neuropathy, epilepsy, dementia, psychiatric disease)

5. Pregnancy or planning pregnancy during the study period

6. Participation in another clinical study that would confound data

7. Unresolved issues of secondary gain (e.g., pending litigation)

### Randomization and allocation concealment

A randomization sequence will be computer generated by the statistician (Qi Zhang) who is not involved in the later statistical work of the study with SPSS 23.0 software, using block randomization with a block size of three or six. An independent researcher will prepare the sealed envelopes to ensure concealment of the allocation sequence. Eligible FBSS patients will be randomly assigned to the EA, MA, or SA group in a 1:1:1 ratio. Only the acupuncturist who performs the treatment will know the group allocation at the time of treatment.

### Blinding

Although it is not practical to blind the acupuncturists to treatment allocations, all of the participants, outcome assessors, and statisticians will be subject to blinding. To achieve successful blinding, a pragmatic placebo needle will be employed and a sham acupuncture design will be applied, and all researchers will be trained before the trial begins.

### Qualification of practitioners

Only licensed acupuncturists with at least 3 years of acupuncture experience will perform the treatment. All the acupuncturists will be instructed in standardized operating procedures prior to the start of the study, such as the location of acupoints and the depth of needling.

### Interventions

**Fig. 2:**
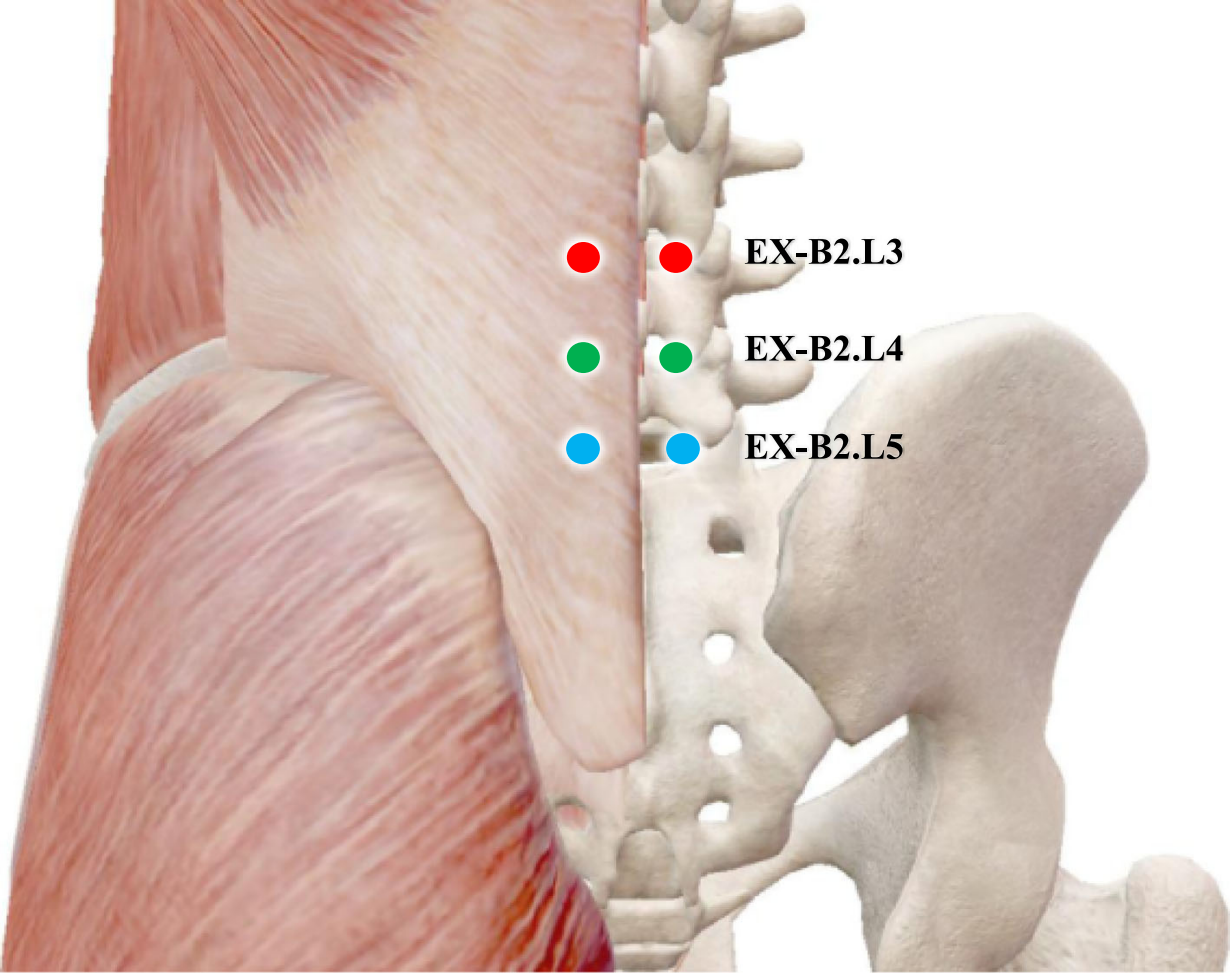
Location of acupoints for FBSS

The interventions will be applied to eligible FBSS patients in three groups for 30 min for 24 sessions over 12 weeks (2 sessions per week). During the treatment, each patient will be placed in a separate quiet space and lying in the prone position, receiving one of the three treatments: electroacupuncture, manual acupuncture, or sham acupuncture. All the acupuncture procedures will follow the Standards for Reporting Interventions in Controlled Trials of Acupuncture (STRICTA) [20]. The location of acupoints for treating FBSS is shown in Fig. 2. In order to improve participants’ adherence, participants will accept the treatment for free and receive financial subsidies (200 RMB) after the last follow-up assessment.

#### The electroacupuncture group

Treatment will be performed using sterilized disposable steel needles (size, 0.35mm × 75mm; Wuxi Jiajian Medical Instrument Co., Ltd, Wuxi, China). Patients will receive acupuncture at bilateral acupuncture points Jiaji (EX-B2. L3, located 0.5cun [≈10mm] lateral to the lower border of the spinous process of the third lumbar vertebra), Jiaji (EX-B2. L4, located 0.5cun [≈10mm] lateral to the lower border of the spinous process of the fourth lumbar vertebra), and Jiaji (EXB2. L5, located 0.5cun [≈10mm] lateral to the lower border of the spinous process of the fifth lumbar vertebra). All acupoints were selected according to the literature [21, 22] and our clinical experience, located with reference to the WHO Standard Acupuncture Locations. After skin disinfection, needles will be inserted perpendicularly for all selected acupuncture points to a depth of 40 to 60mm. After achieving needling sensation [23], an electric stimulator (Huayi BT701-1B; Shanghai Huayi Medical Appliance Factory, Shanghai, China) will be connected to two pairs of needles (EX-B2. L3- EX-B2. L5 bilaterally) to deliver a continuous wave-type pulse at 20-Hz frequency current of 2mA. All needles will be retained for 30 min before removal.

#### The manual acupuncture group

Patients in the manual acupuncture group will undergo similar procedures as the electroacupuncture group except there will be no current output from the electrical apparatus. Sham electrical stimulation technique will be used with the needles connected to functioning electrical stimulators via broken wires.

#### The sham acupuncture group

In the sham acupuncture group, the sham acupuncture device Streitberger placebo needle will be used [24]. The acupoints, electrode placements, and other treatment settings are the same as for the sham electroacupuncture group but with no needling sensation.

### Scheme of analgesic use

The enrolled patients will be allowed to receive celecoxib capsules (Pfizer Pharmaceutica Co. Ltd., New York State, USA) orally on request with a maximum dose of 400 mg/day during the study treatments. The analgesia consumption will be recorded as research data.

### Permitted and prohibited concomitant treatments

During the treatment and follow-up period, participants will not be allowed to initiate any more pain control interventions, which includes but is not limited to other pain blocks, medications, surgeries, and physical therapy. However, general light exercises will be allowed. The researchers will advise the participants to avoid other treatments and re-emphasize at each evaluation time point. Once an individual has utilized other cointerventions, this information should be reported to investigators and recorded for analysis.

### Evaluation

Eligibility was assessed during an initial screening visit. Consent was obtained from all candidates, after which demographic data (age, gender, weight, height, body mass index), duration of pain in months, medical and surgical history, physical examination, segmental level of surgery, and radiographic investigations were taken.

### Outcomes

The primary outcome will be pain intensity of low back. The secondary outcomes will be functional disability, depression symptoms, sleep quality, and analgesic consumption. All outcome data were collected at baseline, during the treatment phase (at 8 and 12 weeks), and at the 16-, 24-, and 36-week follow-ups.

#### Primary outcome measurement

The primary outcome measure is the pain intensity of the low back assessed using an 11-point numerical rating scale (NRS) [9]. Patients will record their low back pain two times per day on a diary for 3 days prior to assessment. The pain intensity for each assessment was calculated as the average NRS available during that period.

#### Secondary outcome measurements

##### Functional disability

The Oswestry Disability Index (ODI) questionnaire will be used to assess functional disability due to FBSS. This questionnaire consists of 10-item scales with 6 response categories each. Each item scores from 0 to 5, which are transformed into a 0 to 100 scale, with scores 0 to 20 have a minimal disability, 21 to 40 have a moderate disability, 41 to 60 have a severe disability, 61 to 80 are crippled, and 81 to 100 are bed-bound or exaggerating their symptoms. A change in ODI of 6% is considered clinically meaningful [25].

##### Depression symptoms

The Beck Depression Inventory-II (BDI-II) is an instrument that assesses the severity of depression. It contains 21 items on a 4-point scale from 0 to 3 and the final score ranges between 0 and 63 points, which reflects the presence of depressiveness and severity thereof. The scores for depression are minimal (<14), mild (14–19), moderate (20–28), and severe (>28). Respondents are instructed to answer the items based on how they have been feeling over the past 2 weeks [26].

##### Sleep quality

FBSS-associated sleep quality disturbance was assessed by the Pittsburgh Sleep Quality Index (PSQI). The PSQI questionnaire is composed of 19 items that yield 7 component scores, each component yields a score ranging from 0 to 3, with 3 indicating the greatest dysfunction. A total score of >5 is identified as disordered sleep [27].

##### Analgesic consumption

Celecoxib will be the only available analgesic, and the total amount was divided by the follow-up period to detect the daily requirement at each follow-up interval. The investigators will also offer free consultation during the trial and use tablets returned every follow-up interval to increase the participants’ compliance.

### Adverse events

Common adverse events (AEs) in acupuncture clinical trials include subcutaneous hematoma, fainting, continuous post-needling pain, and allergies at the sites of needle insertion [28]. Patients with any undesirable effect during the trial will be advised to contact their acupuncturists. The acupuncturists will treat the adverse reactions immediately and contact relevant researchers. All the details (include the time of occurrence, symptoms, duration of symptoms) of AEs will be reported by researchers in the case report forms (CRFs) and the ethics committee immediately. Participants have the rights to take any medical treatment to alleviate uncomfortable symptoms whether it is or not caused by the intervention. The ethics committee of Shanghai Municipal Hospital of Traditional Chinese Medicine takes responsibility for deciding whether to suspend the trial once any AE occurs. After accessing and investigating the cause of adverse events, relevant free medical treatment and appropriate financial compensation will be made to participants.

### Withdrawal

Investigators will evaluate the participants who find their low back pain cannot be alleviated by acupuncture (base on a NRS score ≥6) or have serious AEs (severe infection, coma, shock) to discontinue the study. Furthermore, participants can discontinue the treatment at any time for any reason. All discontinued participants’ data will be treated as missing data.

### Data collection and management

Clinical data will be collected using the printed CRFs, which will be stored securely in a locked cabinet in the office at the hospital. Two independent investigators will enter the data into the clinical trial management platform ResMan and check for double entry to ensure the accuracy of data [29]. All electronic datasets will be de-identified and password protected. Both paper files and electronic documents are accessed to only authorized researchers. After verification, the database will be locked and delivered for statistical analysis. All original CRFs and consent forms will be kept strictly confidential and preserved for 10 years after publication. Only if there exists a life-threatening emergency event when it is a must to know the allocated intervention which participant has received, unblinding is permissible and the researcher can reveal the allocated details in the sealed envelope. Once unblinding occurred, the participant should be discontinued in the trial and the investigator should record the reason for the discontinuation in CRF.

### Monitoring

The trial will be conducted by the Shanghai Municipal Hospital of Traditional Chinese Medicine who will be responsible for coordinating, developing, and controlling the quality of all the programs. For the purpose of monitoring data, an independent Data and Safety Monitoring Board (DSMB) will be set up to review the trial data every month to ensure its accuracy and authenticity. The members of the DSMB who all have declared no conflict of interest in this trial will take the role of evaluating the safety of participants during the trial, monitoring the study progress, guaranteeing the accuracy and completeness of data, and providing recommendations to continuing, modifying, suspending, or terminating the study. Besides, any revisions of the protocol will be discussed by the ethics committee of Shanghai Municipal Hospital of Traditional Chinese Medicine timely.

### Sample size

The trial was aimed to determine whether there was a difference among the EA group, MA group, and SA group in terms of NRS scores. The sample size was calculated based on the previous study [30] through the PASS system (version 15.0.5), with the assumption of the difference in the mean NRS scores between the MA group and SA group as 1.5 and the standard deviation as 2.0. Thirty-nine participants per group would be required to provide 90% power with a two-sided significance level of 5%. Therefore, considering a 20% dropout rate, a total of 144 participants will be recruited eventually in the study.

### Statistical analysis

Statistical analyses will be performed using SAS (version 9.4) by an independent statistician totally blinded to the group allocation. All data will be analyzed based on the intention-to-treat principle, including the missing data from dropouts which will be substituted into the collected data in the last assessment. For measurement data, if the data is in conformity to normal distribution and homogeneity of variance, data values will be presented as mean ± standard deviation (*MD*±*SD*) and covariance analysis will be applied to compare data between groups. If the data does not comply with the normal distribution, median (P25, P75) will be adopted to describe it and the Mann-Whitney *U* nonparametric test will be applied for the comparison between groups. For categorical data, data can be presented as frequency, composition ratio, or rate on the basis of data results. When it comes to dichotomous or unordered multinomial data, the *X*^2^ test will be used to compare the difference between groups, whereas the rank sum test will be adopted to analyze data when it belongs to ordered multinomial value. A two-sided *P* < 0.05 will be considered as significance for all statistical comparisons.

## Discussion

Integration of acupuncture into clinical pain management is expanding rapidly due to the effort of reducing reliance on traditional analgesics. Mounting evidence supports the analgesic effect of acupuncture in the diseases such as low back pain, neck pain, cancer pain et al. [12, 31, 32]. However, a randomized controlled trial (RCT) study about EA relieving FBSS symptoms has not been reported. This trial was designed to investigate the efficacy and safety of EA in patients with FBSS. We hope the results of this study will contribute to clinical practice by providing evidence and provide a low-risk beneficial nondrug therapy for FBSS patients.

Although manual acupuncture and electroacupuncture have obvious therapeutic effects compared to sham acupuncture, the efficacy between manual acupuncture and electroacupuncture that is superior is still controversial [33]. In this study, we separate these two treatments and thus can evaluate the effects separately. We also expect the results of this pilot RCT can provide relevant evidence, which can promote the clinical application of EA as acupuncture analgesia for FBSS.

There are still some limitations and challenges in this study. First, it is impossible to blind the acupuncturists with regard to treatment allocation. Second, due to the sample size, the study may not be able to have enough power to test the difference between manual acupuncture and electroacupuncture. Furthermore, although acupuncture has been demonstrated to be effective in numerous lumbar diseases, FBSS caused by different etiologies may lead to the diversity of acupuncture treatment effects. In a word, we will strive to standardize any step of the study to obtain a high research quality. We hope the results of this study will provide reliable evidence and clarify the therapeutic effect of electroacupuncture in FBSS.

## Trial status

Protocol: version 1.0, 25 October 2020.

The study launched on 31 December 2020. And now participant recruitment is in progress. Participant recruitment and data collection are expected to be completed by the end of December 2022.

## Supplementary Information

**Additional file 1:.** Standard Protocol Items: Recommendations for Interventional Trials (SPIRIT) 2013 checklist: recommended items to address in a clinical trial protocol and related documents.

## Data Availability

The data in this trial will be publicly available from the corresponding author upon reasonable request. All data and the protocol will be available after publication in peer-reviewed international journals for 3 years.

## Abbreviations

AE: Adverse event
BDI-II: Beck Depression Inventory-II
CONSORT: Consolidated Standards of Reporting Trials
EA: Electroacupuncture
FBSS: Failed back surgery syndrome
NRS: Numerical rating scale
ODI: Oswestry Disability Index
PSQI: Pittsburgh Sleep Quality Index
RCT: Randomized controlled trial
SA: Sham acupuncture
SCS: Spinal cord stimulation
MA: Manual electroacupuncture
SPIRIT: Standard Protocol Items: Recommendations for Interventional Trials; Standard protocol items
STRICTA: Standards for Reporting Interventions in Controlled Trials of Acupuncture
TENS: Transcutaneous electrical nerve stimulation
